# Synergistic Effects of Air Pollutants and Extreme Temperature on Asthma: A Narrative Review of Mechanisms and Evidence

**DOI:** 10.3390/toxics14050452

**Published:** 2026-05-21

**Authors:** Guanlin Li, Junliang Chen, Ao Wang, Shunjie Hao, Charles Obinwanne Okoye, Yueru Qiao, Yu Cheng, Hui Liang, Linjing Deng, Xunfeng Chen

**Affiliations:** 1 Key Laboratory of Zhenjiang, School of Environment and Safety Engineering, Jiangsu University, Zhenjiang 212013, China; liguanlin@ujs.edu.cn (G.L.);; 2 School of Emergency Management, Jiangsu University, Zhenjiang 212013, China; 3 Jiangsu Collaborative Innovation Center of Technology and Material of Water Treatment, Suzhou University of Science and Technology, Suzhou 215009, China; 4 Department of Zoology & Environmental Biology, University of Nigeria, Nsukka 410001, Nigeria; 5 Marine Geological Survey of Jiangsu Province, Nanjing 210007, China; 6 School of Management, Jiangsu University, Zhenjiang 212013, China; 7 Shandong Key Laboratory of Environmental Processes and Health, School of Environmental Science and Engineering, Shandong University, Qingdao 266237, China

**Keywords:** asthma, air pollution, temperature, oxidative stress, narrative review

## Abstract

Global climate change and air pollution jointly threaten respiratory health. Asthma, a prevalent chronic inflammatory airway disease, is exacerbated by both traditional air pollutants such as particulate matter (PM_2.5_), ozone (O_3_), nitrogen dioxide (NO_2_), sulfur dioxide (SO_2_) and emerging contaminants like microplastics (MPs) and per- and polyfluoroalkyl substances (PFAS), with effects amplified under extreme temperature conditions. In reality, individuals face complex combined exposures, yet the synergistic effects of these factors on asthma pathogenesis remain poorly understood. This narrative review synthesizes epidemiological and toxicological evidence. It aims to elucidate both the individual and the notably synergistic effects of these factors on asthma pathogenesis. The central mechanistic pathway is initiated by oxidative stress, which activates key inflammatory signaling pathways, thereby driving immune imbalance and airway inflammation. Our review underscores that the combined exposure to traditional pollutants, emerging pollutants, and extreme temperatures may pose a greater threat than individual factors. These findings underscore the critical need for an integrated perspective in asthma research and public health policy. Moving beyond single-pollutant approaches, we advocate for combinatorial risk assessment and synergistic intervention strategies to effectively mitigate the growing burden of asthma in a changing climate.

## 1. Introduction

Air pollution poses a significant health risk in developing countries, contributing to respiratory disease [1], cardiovascular disease [2], reproductive system disease [3], and neurological disease [4], especially in respiratory illnesses. Air pollutants include particulate matter (PM_2.5_), gaseous pollutants such as ozone (O_3_), nitrogen dioxide (NO_2_) and sulfur dioxide (SO_2_), as well as emerging contaminants like microplastics (MPs) and per-and polyfluoroalkyl substances (PFAS). Air pollutants primarily come from energy processes, transport, heating and agriculture. Inhalation of air pollutants impacts the majority of organ systems, eliciting diverse physiological alterations, organ impairment, and clinical disease [5,6,7].

**Figure 1:**
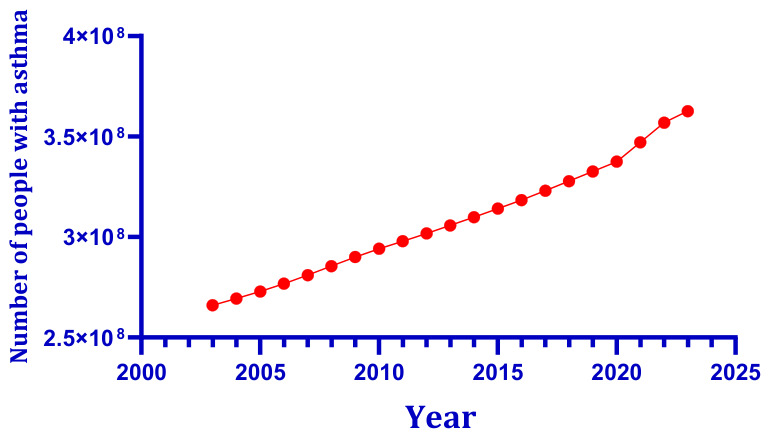
Number of people with asthma, 2003–2023. Between 2003 and 2021, the global prevalence of asthma increased, driven by both population growth and improved case detection. Data for 2003–2021 are from the Global Burden of Disease Study (GBD) [39]. Solid red circles indicate observed annual values.

As a chronic inflammatory condition affecting the bronchial tube, asthma is typified by recurring episodes of coughing, chest tightness, and audible wheezing. It poses a serious health threat to vulnerable populations, including children, pregnant women, and the elderly [8,9,10]. The Global Asthma Report reveals that asthma affects 9.1% of children, 11.0% of teenagers and 6.6% of adults worldwide [11]. The global burden of asthma remains substantial and exhibits complex temporal dynamics over the past two decades. As shown in Figure 1, according to data from the Global Burden of Disease Study (GBD), the number of people with asthma remained generally stable from the early 21st century through 2021 [12]. In stark contrast, the number of deaths across all age groups has been on a steady rise, with the increase concentrated primarily among women and the elderly [12].

There is strong epidemiological evidence linking air pollution to asthma exacerbations. For example, a meta-analysis revealed that increased levels of PM_2.5_ were associated with higher rates of asthma-related emergency room admissions [13]. Another study revealed that brief exposure to O_3_, SO_2_, and NO_2_ significantly increased the likelihood of acute asthma attacks, as evidenced by emergency room visits and hospital admissions [14]. Beyond traditional pollutants, emerging contaminants were also a cause for concern. For instance, some research also indicated that elevated serum PFAS concentrations were significantly associated with a high risk of asthma among Norwegian adolescents [15]. While epidemiological research into the link between MPs and asthma is currently limited, toxicological studies suggest potential harm. These airborne particles originate from diverse sources and have also been detected in agricultural soils, where they alter crop antioxidant defenses and rhizospheric microbiota under fluctuating moisture regimes, pointing to broad environmental persistence and multiple potential routes of human exposure [16,17,18]. Notably, PM_2.5_ has been well-established as an environmental risk factor for asthma.

The pathogenesis of asthma induced by air pollution involves a complex interplay of mechanisms, with oxidative stress serving as a central component. Animal studies have demonstrated that exposure to different pollutants disrupted pulmonary redox homeostasis. For instance, PM_2.5_ exposure markedly reduced the levels of key antioxidants, such as catalase (CAT), glutathione (GSH), and total superoxide dismutase (T-SOD) [19]. Similarly, exposure to O_3_ and NO_2_ elevated reactive oxygen species (ROS), leading to direct airway epithelial damage [20], and increased lipid peroxidation as indicated by elevated malondialdehyde (MDA) alongside decreased GSH [21]. In addition to traditional air pollutants, emerging contaminants, including MPs, can exert toxic effects through oxidative stress. Studies indicated that MPs via the trachea significantly suppressed the activity of the pulmonary antioxidant enzyme SOD, induced alveolar epithelial damage, and subsequently led to pulmonary fibrosis [22,23]. This oxidative stress was intrinsically linked to heightened endoplasmic reticulum (ER) stress, which was directly associated with impaired lung function and correlated with elevated immunoglobulin E (IgE) levels in asthmatic models [24]. Furthermore, specific inflammatory pathways were activated by pollutants, such as the signal transducer and activator of transcription 6 (STAT6)-mediated enhancement of T helper type 2 cell (Th2) responses induced by SO_2_ [25]. Additionally, it was demonstrated that PFAS can directly initiate and activate the NOD-like receptor family, pyrin domain containing 3 (NLRP3) inflammasome in human bronchial epithelial cells. This activation led to the release of the inflammatory mediator caspase-1 and upregulated pro-inflammatory cytokines such as interleukin-6 (IL-6) and interleukin-5 (IL-5) [26]. Moreover, the increased expression of sensory pathways like the transient receptor potential vanilloid 1 (TRPV1) in airway epithelium has been observed in severe asthma [27]. Collectively, these findings suggested that diverse air pollutants converged to disrupt oxidative balance, triggered ER stress, and activated specific inflammatory and sensory pathways, thereby exacerbating asthma.

Relative to the 1901–2000 average baseline maintained by the NOAA National Centers for Environmental Information (NCEI), global mean surface temperature has exhibited a sustained upward trend since the mid-20th century, with the years 2014–2025 constituting the warmest decade in the 175-year instrumental record [28]. Climate change introduces another critical dimension of risk by increasing temperature variability and extreme weather events. In the context of global climate change, higher surface temperatures have exacerbated the occurrence, severity, and persistence of extreme meteorological phenomena [29]. The European heatwave in 2003 was estimated to have caused more than 70,000 deaths [30]. Mean maximum temperatures exceeding 40 °C were recorded in 23 Indian states during the 2020 summer season, according to data from national meteorological agencies [31]. High temperatures increased mortality and acute morbidity from respiratory disease. In addition, heatwaves, wildfires, and droughts associated with high temperature could worsen respiratory diseases [32]. Extreme temperatures, with their wide impact and large affected populations, could aggravate airway hyperresponsiveness (AHR) in asthma patients indirectly [33]. Warmer temperature, when combined with indoor and outdoor air pollutants and allergens, increase the risk of asthma [34]. Furthermore, epidemiological and toxicological evidence established that extreme temperature conditions (heat and cold) exerted significant effects on asthma [35,36,37,38]. These findings suggest that climate change induced extreme weather events pose a severe hazard to human health. Hence, the health problems caused by global climate change should attract widespread attention.

Despite growing evidence on individual factors, a critical knowledge gap remains. In reality, people encounter intricate combinations of conventional and novel air contaminants amid varying temperature conditions. The potential synergistic or additive effects of these combined exposures on asthma pathogenesis, and the underlying integrated molecular mechanisms, are not yet systematically understood.

**Figure 2:**
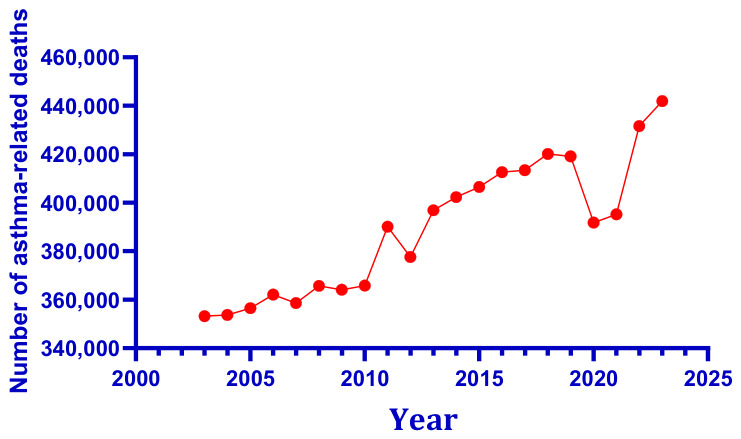
Number of asthma-related deaths, 2003–2023. Over the past two decades, asthma-related deaths have followed a steady upward trend, reaching an estimated 442,000 cases in 2023. The rise in absolute numbers of deaths is concentrated among older age groups and women, primarily reflecting global population aging rather than a deterioration in clinical management. Data for 2003–2021 are sourced from the GBD [39]. Solid red circles indicate observed annual values.

**Figure 3:**
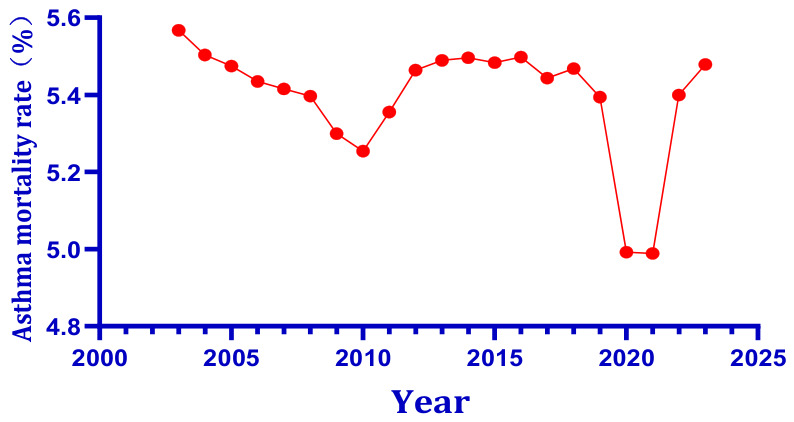
Trends in global age-standardized asthma mortality, 2003–2023. This stands in stark contrast to the rise in absolute mortality. This discrepancy reveals a demographic paradox in asthma mortality: although population aging and growth have led to an increase in absolute mortality, the risk of death in specific age groups has actually declined, suggesting improved disease control per capita. Data for 2003–2021 are sourced from the GBD [39]. Solid red circles indicate observed annual values.

**Figure 4:**
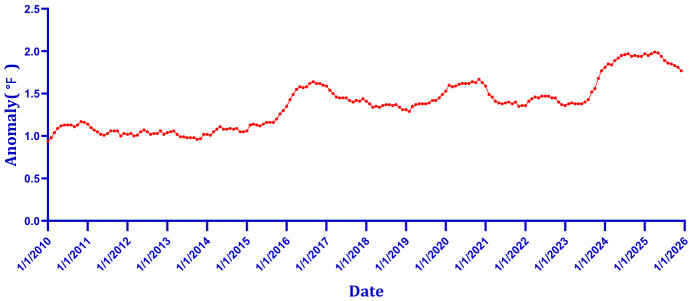
Global annual mean surface temperature anomalies from 2010 to 2026 relative to the 1901–2000 average. The sustained elevation through 2025 and early 2026 confirms an unambiguous long-term warming trajectory, with short-term cyclical fluctuations occurring against an increasingly elevated thermal baseline. Data are derived from the NOAA National Centers for Environmental Information (NCEI) [28]. Solid red circles indicate observed annual values.

As shown in Figure 1, the number of people with asthma worldwide has remained relatively stable since the early twenty-first century. Figure 2 documents a steady upward trend in asthma-related deaths, with the rise concentrated primarily among women and the elderly, reflecting global population aging rather than a deterioration in clinical management per se. In parallel, Figure 3 illustrates that global asthma prevalence, and the proportion of the population affected, has followed a dynamic trajectory driven by population growth and improved case detection. Against this epidemiological backdrop, global climate change introduces an additional layer of complexity. Figure 4 presents the sustained elevation of global mean surface temperature anomalies through 2025 and early 2026, confirming an unambiguous long-term warming trajectory relative to the 1901–2000 baseline. The convergence of these trends, stable asthma case numbers amid rising absolute mortality but declining age-specific risk, alongside an increasingly elevated thermal baseline, underscores the urgent need to examine how combined environmental stressors, rather than single agents, shape respiratory disease burden in a changing climate.

In this review, we synthesize current evidence on the impacts of air pollutants and temperature on asthma, along with the associated molecular mechanisms. Based on this synthesis, we propose corresponding prevention strategies for vulnerable populations and management recommendations for policymakers. We specifically focus on elucidating the potential interactions among these environmental factors, as well as the mechanisms linking them to asthma. The findings aim to provide a scientific basis for asthma prevention. Thus, curbing emissions and addressing extreme temperatures are critical for reducing asthma incidence and enhancing quality of life.

## 2. Sources and Selection Criteria

### 2.1. Search Strategy

Literature searches were systematically conducted in PubMed and Web of Science databases. The search strategy combined keywords related to environmental exposures and asthma outcomes, including but not limited to: “asthma”, “air pollution”, “particulate matter”, “ozone”, “nitrogen dioxide”, “sulfur dioxide”, “microplastics”, “per- and polyfluoroalkyl substances”, “temperature”, “heatwave”, “extreme heat”, “cold”, “climate change”, “oxidative stress”, “inflammation”, and “mice”. These terms were combined using Boolean operators (AND, OR) to comprehensively capture studies on both individual and combined environmental exposures related to asthma.

### 2.2. Study Selection and Evidence Prioritization

A two-stage screening process was applied: initially, irrelevant literature was excluded based on titles and abstracts, followed by full-text evaluation of remaining studies. Included research had to meet two fundamental criteria: (1) the topic directly addressed the epidemiological or mechanistic association between air pollutants or temperature and asthma; (2) and the study was published in a peer-reviewed academic journal.

**Figure 5:**
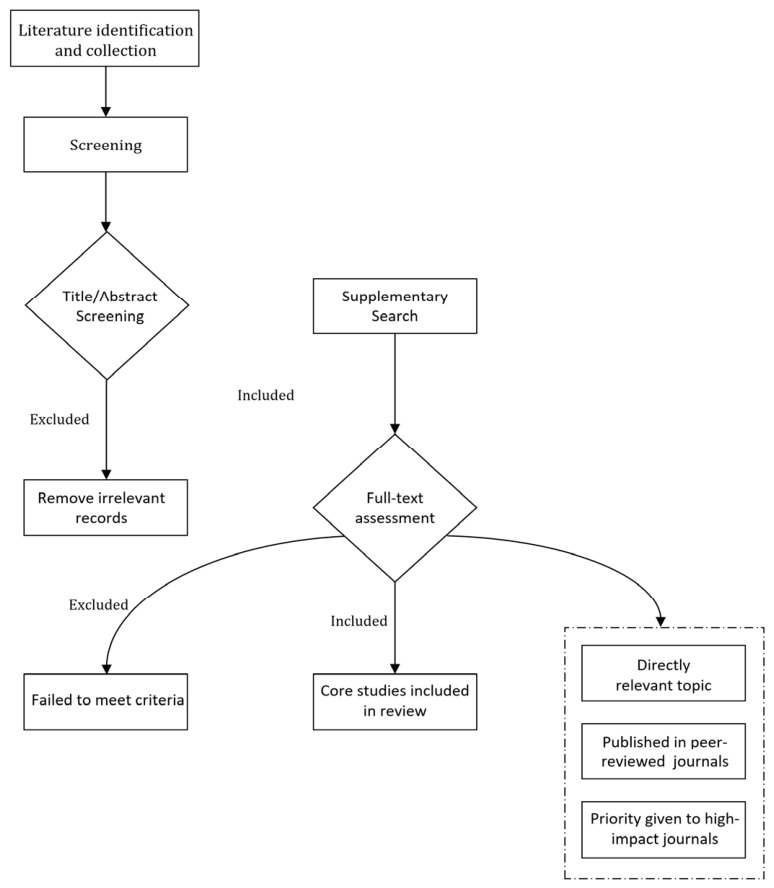
Literature inclusion flowchart.

Given the narrative nature of this review and the marked heterogeneity in evidence maturity across exposure categories, studies were prioritized according to the following principles. For classical pollutants (PM_2.5_, O_3_, NO_2_, SO_2_), priority was given to large-scale cohort studies, meta-analyses, and multi-city investigations that established robust exposure–response relationships. For mechanistic toxicology, priority was given to in vivo and in vitro studies with explicit pathway delineation, with particular emphasis on studies elucidating oxidative stress, inflammatory signaling, and immune polarization, given their direct relevance to the proposed integrative framework. For emerging contaminants (PFAS and MPs), studies with asthma-specific endpoints were favored over general respiratory toxicity reports, given the currently limited and developing evidence base; where direct epidemiological data were scarce, toxicological studies providing mechanistic plausibility were selectively incorporated with explicit acknowledgment of their preliminary status. For combined pollutant–temperature effects, experimental studies employing controlled co-exposure designs and epidemiological analyses with explicit interaction testing were prioritized, reflecting both real-world exposure complexity and the statistical power required to detect genuine interactions. This selection workflow is summarized in Figure 5.

### 2.3. Scope and Limitations

As a narrative review, this paper did not conduct a systematic, exhaustive literature search nor did it use standardized tools to assess the risk of bias in each study. The absence of a predefined search protocol and duplicate screening may introduce selection bias. This review aims to selectively synthesize and interpret existing evidence based on the authors’ understanding of the field, thereby constructing a logical framework for the synergistic effects of pollutants and temperature and identifying future research directions. This article has inherent methodological limitations. These limitations are explicitly addressed throughout the manuscript, particularly in sections where stronger claims are made regarding emerging contaminants and combined exposures.

## 3. Epidemiological Studies on Environmental Factors and Asthma

### 3.1. PM_2.5_

#### 3.1.1. Short-Term and Long-Term Exposure Effects

Epidemiological evidence consistently indicates that both short-term fluctuations and sustained elevations in PM_2.5_ are associated with adverse asthma outcomes, though effect sizes vary by exposure duration, region, season, and population susceptibility periods [40,41,42].

Short-term exposure is linked to increased asthma exacerbations, emergency department visits, and hospitalizations; a meta-analysis demonstrated that a 10 μg/m^3^ increment in ambient PM_2.5_ was associated with elevated childhood asthma hospitalization risk, with particularly pronounced effects during warmer seasons, suggesting possible temperature-mediated modulation of particulate toxicity [13,43]. Large-scale time-series studies in Beijing involving over 90,000 asthma patients have quantified same-day associations between PM_2.5_ increments and outpatient visits [44]. Notably, even within lower concentration ranges (4.00–7.06 μg/m^3^), each per-unit increase in PM_2.5_ remained associated with elevated symptom prevalence, indicating a lack of evident threshold for susceptible individuals [42]. Beyond these acute effects, chronic PM_2.5_ exposure has been independently associated with incident asthma in both children and adults [45,46], as well as with prolonged asthma-related hospitalization duration among pediatric patients, reflecting more severe disease progression [47].

#### 3.1.2. Susceptibility and Exposure Heterogeneity

The epidemiological profile of PM_2.5_-associated asthma risk exhibits substantial heterogeneity across demographic, environmental, and compositional contexts. Older adults demonstrate heightened vulnerability to short-term fluctuations [48], whereas children show stronger responses to sustained exposure [49]. Stratified analyses revealed disproportionate burdens among populations residing in commercial areas, females, Hispanic and Black individuals, and children aged 2–5 years, underscoring the intersection of environmental exposure, socioeconomic context, and intrinsic susceptibility in shaping PM_2.5_-related asthma risk [50]. Seasonal patterns further complicate exposure–response relationships: the concordance of elevated PM_2.5_ with higher ambient temperatures and increased photochemical activity during warm seasons may amplify oxidative and inflammatory stress in airways [13,42], a pattern consistent with the proposed interaction framework.

Direct evidence for temperature modification of PM_2.5_ effects has been demonstrated in a large U.S. survey of 50,356 adults with active asthma, where the association between 14-day average PM_2.5_ and symptom prevalence varied significantly across temperature quintiles [42]. The strongest effects were observed at temperature extremes: in the coldest range (1.1–44.4 °F, −17.2 to 6.8 °C), each 1 μg/m^3^ increase in PM_2.5_ within the 4.00–7.06 μg/m^3^ range was associated with a 7.9% increase in symptom prevalence, while in the moderate–high range (70.2–80.5 °F, 21.2–26.9 °C), the corresponding increase was 7.3% [42].

Cold-season PM_2.5_ effects have been further characterized in Fresno, California, a region with persistent wintertime pollution due to temperature inversions and poor dispersal conditions [50]. During November through February, cumulative 14-day PM_2.5_ exposure demonstrated a clear dose–response relationship with asthma emergency department visits, with statistical significance emerging at 9 μg/m^3^ and increasing progressively at higher concentrations [50]. These findings suggest that both cold stress and heat stress may potentiate PM_2.5_-induced airway inflammation. Such temperature-dependent modulation of particulate toxicity, ranging from wintertime inversion-trapped particulates to warm-season photochemical enhancement, provides epidemiological grounding for the interaction framework developed in the subsequent discussion, where thermal extremes and PM_2.5_ are hypothesized to converge on shared molecular targets.

### 3.2. O_3_

#### 3.2.1. Short-Term and Long-Term Exposure Effects

O_3_ exposure has been consistently associated with both acute asthma exacerbations and chronic lung function decline [51,52,53,54]. The hazards of ozone exposure were evident in both short-term and long-term exposure scenarios. Short-term acute exposure primarily triggered worsening asthma symptoms and acute exacerbations. Studies have confirmed that acute O_3_ exposure can rapidly lead to increased asthma symptoms [51], and demonstrated a significant correlation with heightened risks of emergency room visits for asthma [14]. A nationwide study further quantified its immediate impairment of lung function, finding that for every 4.9 ppb increase in warm-season O_3_ concentration, forced expiratory flow decreased by 14.2 mL/s [52].

Even more serious risks were posed by long-duration chronic exposure. It has been established as an independent factor that precipitates new asthma cases. Multiple cohort studies have indicated that prolonged exposure to O_3_ during childhood significantly increased the incidence of asthma [53,54]. Additionally, long-term exposure also drove progressive lung function impairment and small airway dysfunction. Adult-onset exposure not only triggered acute exacerbations and damaged lung function [55], but also correlated with reductions in average expiratory flow rate, as demonstrated in nationwide studies [52].

These associations, while robust across multiple geographic settings, demonstrate variability related to urbanization level and baseline air quality [56], reflecting the importance of local environmental context and potential modification by co-occurring pollutants and meteorological conditions.

#### 3.2.2. Susceptibility and Exposure Heterogeneity

The respiratory toxicity of O_3_ is heightened in children and individuals with pre-existing AHR [53,54]. Epidemiologically, peak ambient O_3_ concentrations coincide with high temperature and intense solar radiation during warm seasons, creating photochemical smog conditions [57]. Systematic review evidence further confirms that temperature modifies the respiratory health effects of ambient oxidants, with combined heat and O_3_ exposures producing greater deficits than either stressor alone [58]. Review evidence further elucidates that heat stress increases ventilation rates and pollutant uptake while lowering the threshold for bronchoconstriction, thereby potentiating the inflammatory and oxidative airway injury induced by photochemical oxidants [59]. A longitudinal study of adults with asthma demonstrated that ambient O_3_ adversely affects peak expiratory flow, and that this effect is modified by lower ambient temperatures, which amplify O_3_-induced lung function decrements [60]. These seasonal patterns support the hypothesis that O_3_-induced asthma morbidity is not merely a function of ozone concentration but is modified by concurrent temperature. This epidemiological modification of oxidant toxicity by ambient temperature foreshadows the mechanistic interactions between pollutants and thermal stress that are examined at the cellular and systemic levels in the subsequent discussion.

### 3.3. NO_2_

#### 3.3.1. Short-Term and Long-Term Exposure Effects

Epidemiological evidence identifies ambient NO_2_ as a critical environmental risk factor for asthma initiation and deterioration. Short-term exposure to NO_2_ is primarily associated with acute exacerbations of asthma symptoms. A 2021 meta-analysis confirmed a dose–response relationship between short-term NO_2_ exposure and acute asthma attacks [14], establishing it as a recognized risk factor for moderate-to-severe asthma in both children and adults [61]. In multi-city studies involving Chinese cohorts, short-term NO_2_ exposure correlated positively with higher asthma outpatient attendance, with pediatric populations showing greatest susceptibility; approximately 10.9% of asthma outpatient visits could be attributed to NO_2_ exposure [62]. Moreover, heightened indoor NO_2_ levels in metropolitan areas demonstrated significant positive correlations with elevated childhood asthma incidence [63,64,65]. A recent nationwide case-crossover study covering 20 provinces in China provided refined quantification: at lag 0–1 days, every 10 μg/m^3^ increase in NO_2_ was significantly associated with a 4.95% (95% CI: 2.93–6.97%) increase in childhood asthma hospitalization risk, with NO_2_ demonstrating the strongest and most robust independent association in two-pollutant models [66].

Traffic-related NO_2_ exposure over extended periods substantially raised both incident risk of asthma and hospitalization rates. Chronic exposure to combustion-related pollution from fossil fuel sources, including motor vehicle emissions, was associated with adult-onset asthma [67]. Among older adults, long-term exposure to vehicular emissions elevated the likelihood of hospital admissions for asthma, with those who had a history of asthma hospitalizations being particularly vulnerable [68]. A 2025 meta-analysis encompassing 40 studies on adolescent asthma quantified this long-term effect: NO_2_ per 10 μg/m^3^ increment was significantly associated with asthma risk (adjusted odds ratio (aOR) = 1.18, 95%, confidence interval (CI): 1.08–1.29), with the association remaining consistent in cohort studies, long-term exposure subgroups, and after publication bias correction [69].

#### 3.3.2. Susceptibility and Exposure Heterogeneity

Population susceptibility to NO_2_ exhibits marked heterogeneity. A 2024 systematic review for the EAACI guidelines confirmed that short-term NO_2_ exposure probably increases asthma-related hospital admissions and emergency department visits, with children, the elderly, and individuals with pre-existing asthma identified as the most vulnerable subgroups [70]. During winter months, reduced natural ventilation and increased use of unvented gas heaters elevate indoor NO_2_ concentrations; a global systematic review identified unvented combustion appliances as major indoor sources and demonstrated that indoor NO_2_ levels are typically associated with respiratory symptoms, particularly in children [71]. A nationwide time-series analysis in Madrid further revealed that during cold months, NO_2_ was the sole chemical air pollutant significantly associated with asthma-related hospital admissions, accounting for 1.5% of attributable cases, with children under 14 years showing pronounced vulnerability [72]. The seasonal concentration of NO_2_ effects during winter months, when indoor exposure peaks and cold stress co-occurs, aligns with the combined exposure biology elaborated in the subsequent discussion.

### 3.4. SO_2_

#### 3.4.1. Short-Term and Long-Term Exposure Effects

Epidemiological evidence underscored SO_2_ as a significant environmental factor in both exacerbating existing asthma and contributing to its fatal outcomes. A 2021 systematic review and meta-analysis commissioned by the World Health Organization found that short-term increases in ambient SO_2_ were significantly associated with respiratory mortality, with high certainty of evidence; concentration–response functions showed linear behavior with no evident threshold, and the authors concluded that the epidemiological evidence supports a causal relationship [73].

Short-term exposure to environmental SO_2_ was strongly associated with acute asthma exacerbations. Meta-analyses reveal that exposure to short-term SO_2_ significantly elevates the likelihood of asthma-related emergency department presentations and hospital admissions [14]. Recent studies further corroborated the link between SO_2_ exposure and heightened asthma morbidity as well as symptom worsening [74,75,76]. Quantitative studies targeting the Chinese population further indicated that for every 10 μg/m^3^ increase in ambient SO_2_ concentration, the excess risk of asthma-related mortality rose by 7.78% [77]. However, evidence specifically linking acute SO_2_ exposure to asthma mortality remained limited and inconsistent, highlighting a need for further investigation [78,79].

Compared to short-term effects, long-term exposure to SO_2_ was more clearly associated with asthma mortality. Population-based investigations revealed a concentration–effect relationship between long-term SO_2_ exposure and asthma-related mortality [74]. While the body of literature remains somewhat limited, current findings suggest that chronic SO_2_ exposure may be associated with heightened risks of fatal asthma outcomes [80,81]. However, a 2025 meta-analysis encompassing 40 studies on adolescent asthma found that the pooled estimate for long-term SO_2_ did not reach statistical significance (OR = 0.99), accompanied by 53.6% heterogeneity; small subgroup sample sizes, unit conversion discrepancies, and conflicting effect directions limit certainty, and an association cannot be excluded [69].

#### 3.4.2. Susceptibility and Exposure Heterogeneity

Temperature extremes modify the respiratory effects of SO_2_. In Chengdu, China, the adverse effects of ambient SO_2_ on acute mortality depended on temperature: a 10 μg/m^3^ increment in SO_2_ increased non-accidental deaths by 1.4% (95% CI: 0.5–2.4%) during high-temperature days (22.8–29.4 °C), compared with 0.8% (95% CI: 0.1–1.5%) during low-temperature days (−0.3–9.3 °C). This suggests that temperature acts as a critical effect modifier for SO_2_-related health outcomes [82]. In South Korea, a time-trend controlled case-crossover study of refractory asthma patients demonstrated that during winter, a 1 ppb increase in SO_2_ concentration at lag 1 day was associated with a 19.7% (95% CI: 3.3–38.7%) increase in the risk of acute exacerbation among nonsmokers, while a 1 °C decrease in ambient temperature at the same lag was associated with a 14.8% (95% CI: 0.9–26.7%) increase [83]. These findings indicate that SO_2_-related asthma risk is amplified under both cold stress and high-temperature conditions, although the underlying exposure pathways and vulnerable populations may differ. The dual temperature modulation of SO_2_ toxicity observed here, enhanced acute effects during heat and exacerbation during cold, mirrors the integrated pollutant–temperature framework examined in the later sections.

### 3.5. Emerging Contaminants

The epidemiological evidence base for emerging contaminants remains markedly uneven, with substantial differences in maturity between MPs and PFAS.

#### 3.5.1. MPs

At present, population-based research directly investigating associations between microplastic exposure and asthma development remained scarce. Most evidence is derived from toxicological experiments, in vitro studies, indirect environmental exposure data, or observational research on other respiratory diseases such as pneumonia and chronic obstructive pulmonary disease (COPD) [84,85,86,87]. A 2023 systematic review noted the scarcity of population cohort studies and concluded that current evidence remains preliminary and insufficient to establish firm conclusions regarding respiratory morbidity in humans [88]. Although airborne MPs have been detected in human blood, lungs, liver, brain, kidneys, and testicles [22,89,90,91,92,93], their presence indicates exposure and biopersistence but does not, by itself, demonstrate a causal contribution to asthma pathogenesis. Occupational studies link inhalable plastic fibers to interstitial lung disease and lung cancer [94,95], yet these findings are not asthma-specific. Consequently, the link between MP exposure and asthma should presently be regarded as a hypothesis supported by toxicological plausibility but not by established population-level evidence.

The current evidence base does not yet allow a clear distinction between short-term and long-term effects of MPs on asthma, because longitudinal cohort studies with defined exposure windows are lacking. Moreover, current toxicological evidence largely derives from controlled laboratory conditions that do not replicate the complex, chronic inhalation patterns typical of real-world human exposure [96,97,98]. Furthermore, commonly employed non-inhalation exposure routes in animal models, such as tracheal instillation, poorly simulate the chronic, low-dose inhalation patterns typical of ambient human exposure. Consequently, the link between microplastic exposure and asthma should presently be regarded as a hypothesis supported by toxicological mechanism but not by established population-level evidence.

#### 3.5.2. PFAS

Studies have reported higher PFAS concentrations in children’s serum compared to adults, confirming that this high-risk population has greater PFAS exposure [99,100]. A genetic and biomarker study of Taiwanese children found that serum concentrations of perfluorooctanoic acid (PFOA), perfluorooctanesulfonic acid (PFOS), and perfluorodecanoic acid (PFDA) were positively correlated with asthma [101,102]. The National Health and Nutrition Examination Survey (NHANES) study of adolescents in the United States also reported a positive correlation between asthma and elevated serum concentrations of PFOA and PFOS [103]. Several other studies similarly have supported a positive correlation between PFAS concentrations and asthma or various allergic diseases [104,105,106,107].

Large-scale cohort studies have strengthened this evidence base while simultaneously revealing complexity. In the Norwegian Tromsø study of 675 adolescents, total PFAS serum concentration in the highest quartile was associated with approximately 3.35 times higher odds of asthma (95% CI: 1.54–7.29) compared with the lowest quartile; notably, total PFOS, linear PFOS, and linear perfluorohexane sulfonate (PFHxS) in the upper quartiles were associated with roughly twofold higher asthma odds, and these associations persisted at three-year follow-up [15]. Complementing these findings, a 2026 Swedish register-based cohort study in Ronneby found that very high prenatal PFAS exposure was associated with a higher incidence of childhood asthma (hazard ratio (HR): 1.44, 95% CI: 1.08–1.92); however, this association was restricted to the extreme exposure category and was not observed at lower exposure levels, indicating a possible threshold effect rather than a linear dose–response [108].

However, this association exhibited certain complexities and specificities. First, the correlation varied depending on the specific type of PFAS, demonstrating subtype-specific effects. For example, research has demonstrated that elevated serum concentrations of PFOA correlate with asthma exacerbations among adolescent populations [109], while other research found that exposure to certain PFAS was linked to reduced lung function [107,110]. Second, the effects may vary across different age groups, exhibiting age-specific effects. A single investigation demonstrated that pediatric subjects with elevated PFOS levels exhibited greater asthma occurrence relative to older age groups [111], while other studies primarily observed positive associations in older children or adolescents [101,103,105]. Sex and exposure-timing differences further complicate the epidemiological profile. A 2016 study of Taiwanese children reported that PFAS associations with T-helper cell cytokines differed by gender and asthma status [102]. Recent analyses of the Japan Environment and Children’s Study found that prenatal exposure to per- and polyfluoroalkyl substances was associated with wheezing and asthma symptoms in 4-year-old children, underscoring early-life vulnerability [112].

Several limitations qualify the above epidemiological patterns. For PFAS, most investigations have examined individual compounds such as PFOA and PFOS, whereas human exposure entails complex real-world mixtures; the extent to which mixture effects differ from those of single agents remains undefined. For MPs, population-level cohort studies with defined exposure windows are currently lacking, and claims regarding asthma causality should therefore be regarded as preliminary. A synopsis of selected epidemiological studies documenting the pollutant–asthma exposure response relationships discussed above is provided in Table 1.

## 4. Toxicological Research on Environmental Factors and Asthma

### 4.1. PM_2.5_

#### 4.1.1. Cellular Experiments

In human alveolar epithelial A549 cells, PM_2.5_ exposure induced intracellular ROS accumulation and triggered apoptotic cell death, whereas overexpression of serum/glucocorticoid-regulated kinase 1 (SGK1) attenuated both oxidative stress and apoptosis, identifying SGK1 as a protective modulator against PM_2.5_-driven epithelial injury [120]. Short-term exposure of A549 cells to dustfall PM_2.5_ further caused G2/M cell cycle arrest and dose-dependent lipid peroxidation, confirming that redox imbalance is an early event in PM_2.5_ cytotoxicity [121]. Using an air–liquid interface exposure system, Yan et al. exposed A549 cells to PM_2.5_ collected from residential, industrial, and traffic areas in Nanjing (25–100 μg, 4 h). Traffic-source particles exerted the strongest cytotoxicity, inducing ROS generation, apoptosis, DNA damage, and elevated IL-6 release [122].

Seasonal PM_2.5_ from high-traffic areas in Karaj and Fardis reduced A549 viability (25–100 μg/mL, 12 h–2 days) and increased IL-6 and interleukin-8 (IL-8) release in a concentration- and time-dependent manner. Notably, winter samples induced approximately twofold higher cytokine responses than summer samples [123].

Collectively, these cellular findings point to a consistent oxidative–inflammatory cascade. PM_2.5_-generated ROS disrupts redox homeostasis and compromises epithelial barrier integrity, while the concurrent rise in IL-6 and IL-8 reflects active pro-inflammatory signaling that promotes immune cell recruitment. Importantly, the same ROS and inflammatory signaling nodes disturbed by PM_2.5_ are also vulnerable to extreme temperature and other air pollutants. This overlap in molecular targets provides a plausible explanation for the synergistic interactions discussed later in this paper.

#### 4.1.2. Animal Experiments

Representative animal studies characterizing PM_2.5_-induced allergic airway inflammation and structural remodeling are summarized in Table 2. Evidence from mouse models demonstrates that PM_2.5_ aggravates allergic airway inflammation and drives structural remodeling through multiple convergent pathways. In C57BL/6 mice, intratracheal instillation of PM_2.5_ (5 mg/kg body weight, every two days for four weeks) in the context of influenza virus challenge downregulated lysine demethylase 6a (Kdm6a) expression in pulmonary macrophages, increased H3K4 and H3K9 methylation at the IL-6 and interferon-beta (IFN-β) promoter regions, and disrupted immune homeostasis through epigenetic reprogramming [124].

In Swiss mice (male, 8 weeks old), intranasal instillation of PM_2.5_ (2.5 or 5 μg, single or repeated doses) induced acute oxidative stress, evidenced by elevated MDA and altered antioxidant enzyme activity, alongside pulmonary inflammation characterized by neutrophil infiltration and increased IL-6 and tumor necrosis factor-alpha (TNF-α) levels in bronchoalveolar lavage fluid (BALF) [129]. In ovalbumin (OVA)-sensitized BALB/c mice, ambient PM_2.5_ exposure significantly enhanced allergic airway inflammation, as shown by increased inflammatory cell infiltration, mucus hypersecretion, and AHR. Co-exposure with OVA further elevated Th2 cytokines and decreased IFN-γ, indicating Th1/Th2 immune imbalance. Concurrently, PM_2.5_ upregulated the protein expression of transient receptor potential ankyrin 1 (TRPA1) and TRPV1 in lung tissue [125]. In a separate OVA-sensitized BALB/c model, urban PM_2.5_ (10 mg/kg, intratracheal instillation, twice weekly for four weeks) exacerbated allergic lung inflammation via activation of the TLR2/TLR4/MyD88 signaling pathway, driving downstream nuclear factor kappa-B (NF-κB)-mediated inflammatory responses [128].

These PM_2.5_-induced disturbances in redox signaling and chromatin remodeling prime the airway epithelium for exaggerated responses when concurrent thermal stress or other pollutants further challenge the same molecular targets. In human asthma, the same molecular targets, redox-sensitive signaling cascades and chromatin-modifying enzymes, are activated in severe disease, suggesting that the experimental perturbations described here translate to clinically relevant mechanisms of airway sensitization [130].

### 4.2. O_3_

#### 4.2.1. Cellular Experiments

In vitro evidence demonstrates that O_3_ directly injures respiratory epithelial cells through oxidative and inflammatory mechanisms. In human bronchial epithelial BEAS-2B cells, ozone exposure (0.16 mg/m^3^) time-dependently increased the secretion of interleukin-1 (IL-1) and IL-6, elevated intracellular MDA, and generated ROS, indicating that low-level O_3_ is sufficient to trigger pro-inflammatory and oxidative stress responses in airway epithelium [131]. In human alveolar epithelial cells, O_3_ induced apoptosis and cytotoxicity in type I-like (ATI-like) cells more markedly than in type II (ATII) cells, accompanied by ROS generation and DNA damage; these effects were mimicked by oxysterols, suggesting that lipid peroxidation products mediate O_3_-induced epithelial injury [132].

#### 4.2.2. Animal Experiments

Key animal experiments examining O_3_-driven airway inflammation, oxidative stress, and AHR are presented in Table 3. In C57BL/6 mice, acute O_3_ inhalation (1 ppm, 1 h) disrupted respiratory epithelial barriers, caused alveolar protein exudation, recruited neutrophils into BALF, and increased AHR to methacholine. Chronic episodic exposure (1.5 ppm, 2 h, twice weekly for 6 weeks) amplified these inflammatory responses and additionally produced peribronchial collagen deposition, enlarged alveolar spaces, and emphysema-like structural changes with epithelial thinning [20].

O_3_-driven airway inflammation is tightly linked to oxidative stress and mitogen-activated protein kinase (MAPK) signaling. In C57BL/6 mice, O_3_ (2.5 ppm, 3 h) increased BALF total cells, neutrophils, and macrophages, elevated TNF-α, IL-6, IL-1β, and MDA, and decreased the reduced/oxidized glutathione (GSH/GSSG) ratio. Preventive and therapeutic treatment with the hydrogen sulfide donor NaHS (2 mg/kg, intraperitoneally) attenuated these changes, restored redox balance, and inhibited p38 MAPK and heat shock protein 27 phosphorylation [133]. In OVA-sensitized BALB/c mice, O_3_ (2.5 ppm, 3 h) similarly enhanced AHR and pulmonary inflammation through p38 MAPK activation and oxidative stress [134].

Together, these findings show that ozone damages airway epithelium through ROS and lipid peroxidation, triggering IL-1 and IL-6 release. O_3_-driven lipid peroxidation and p38 MAPK activation converge with the oxidative–inflammatory mechanisms of particulate matter and heat stress, suggesting that co-exposure amplifies airway injury through shared biochemical lesions rather than independent pathways. These shared biochemical lesions, lipid peroxidation and MAPK-driven inflammation, are detectable in human asthmatic airways exposed to photochemical oxidants, supporting the translational relevance of the murine findings to population-level ozone morbidity [135].

**Table 3:** Research of animal experiments about the relationship between O_3_ and asthma.

Animal Model	Results	Method	Exposure	Reference
C57BL/6 mice	Increases BALF inflammatory cells in a concentration-dependent manner.	Inhalation method	2.5 ppm; 3 h	[133]
C57BL/6 mice	Increases AHR and airway resistance, decreases lung compliance in OVA-asthmatic mice.	Inhalation method	1.0 ppm; 3 h, 23/25/27 day during OVA challenge	[134]
BALB/c mice	Increases IgE and Th2 cytokines with repeated exposure.	Inhalation method	1.0 ppm; 3 h during OVA challenge	[136]
BALB/c mice	Upregulates IL-17A and drives neutrophilic infiltration.	Inhalation method	2.5 ppm; 3 h, twice weekly for 6 weeks (12 total)	[137]
C57BL/6 mice	Destroys respiratory epithelial cells, induces protein exudation and neutrophil recruitment, triggering inflammation and AHR.	Inhalation method	1/1.5 ppm; 1 h/2 h, twice weekly for 6 weeks	[20]

Note: bronchoalveolar lavage fluid (BALF); airway hyperresponsiveness (AHR); ovalbumin (OVA); mitogen-activated protein kinase (MAPK); interleukin (IL); tumor necrosis factor-alpha (TNF-α); reduced/oxidized glutathione ratio (GSH/GSSG).

### 4.3. NO_2_

#### 4.3.1. Cellular Experiments

Human bronchial epithelial cells mount a rapid pro-inflammatory transcriptional response upon direct NO_2_ challenge. In confluent BEAS-2B cultures exposed to 1.5 ppm NO_2_ for 1 h, IL-6 and IL-8 mRNA abundance increased by 23.4-fold and 30.9-fold respectively, with corresponding elevations in secreted protein levels [138]. Concurrent with this inflammatory activation, NO_2_ compromises physical barrier integrity. In primary nasal mucosa cells cultured at the air–liquid interface, NO_2_ exposure decreased transepithelial electrical resistance, reduced occludin mRNA expression, and elevated IL-6 and IL-8, confirming direct tight junction disruption at the upper respiratory gateway [139]. At the alveolar level, direct dynamic exposure of A549 cells to NO_2_ at 2.5–15 ppm produced concentration-dependent cytotoxicity [140]. Collectively, these studies establish that NO_2_, at concentrations relevant to both ambient and occupational settings, directly activates epithelial pro-inflammatory programs, disrupts barrier function, and induces cytotoxicity in human airway cells.

#### 4.3.2. Animal Experiments

In Wistar rats, whole-body inhalation of NO_2_ (2 mg/m^3^ or 5 mg/m^3^, 5 h/day, 28 or 42 days) in OVA-sensitized animals increased inflammatory cell infiltration, mucus hypersecretion, and collagen deposition in lung tissue, indicating heightened allergic airway inflammation and asthma susceptibility [141]. In C57BL/6 mice, exposure to 25 ppm NO_2_ (6 h/day, 3 or 5 days) induced acute lung injury with elevated BALF protein, lactate dehydrogenase (LDH), and neutrophils; when combined with OVA sensitization, this exposure markedly amplified eosinophilic inflammation, terminal bronchiolar lesions, and AHR [142]. In OVA-sensitized BALB/c mice, NO_2_ inhalation (10 ppm, 1 h/day, 7 days) augmented allergic airway inflammation and AHR, with increased eosinophil and lymphocyte infiltration, elevated serum IgE, and upregulated lung tissue IL-4, IL-5, IL-13, and IFN-γ. These effects were accompanied by NF-κB activation in airway epithelium and correlated with enhanced airway smooth muscle mass [143]. Short-term NO_2_ exposure (20 ppm, 1 h/day, 4 days) in BALB/c mice interfered with allergic airway responses to OVA challenge, altering inflammatory cell recruitment and mediator release [144].

These findings trace a clear path from epithelial injury to allergic airway disease. NO_2_ first damages lung epithelium through reactive nitrogen species and density-dependent cytotoxicity, then amplifies Th2-polarized inflammation via NF-κB activation, IL-4/IL-5/IL-13 upregulation, and IgE production, ultimately producing mucus hypersecretion, collagen deposition, and sustained AHR. In human asthma, equivalent pathways, including epithelial barrier disruption, Th2 skewing, and smooth muscle remodeling, represent well-established disease mechanisms [145]. The experimental activation of these shared nodes by NO_2_ therefore provides a plausible biological substrate for the epidemiological associations observed in the earlier discussion, and primes the airway for exaggerated responses when thermal stress or other pollutants concurrently engage the same signaling axes, as discussed in the subsequent discussion.

### 4.4. SO_2_

#### 4.4.1. Cellular Experiments

Direct in vitro exposure studies with SO_2_ gas are markedly scarcer than those for NO_2_, reflecting a persistent evidence gap in the literature [146]. In one of the few available dynamic direct-exposure investigations, A549 alveolar epithelial cells cultured at the air–liquid interface were exposed to SO_2_ concentrations from 10 ppm to 200 ppm, resulting in a concentration-dependent reduction [140]. Because of the limited direct-gas cellular literature, mechanistic understanding of SO_2_-induced airway injury has historically relied on derivative models. In human bronchial epithelial BEP2D cells treated with sodium bisulfite and sodium sulfite (1:3 ratio, 0.0001–1 mM) for up to 24 h, epidermal growth factor (EGF) and epidermal growth factor receptor (EGFR) mRNA and protein increased in a dose-dependent manner, peaking at 30 min; this pattern was predicted to drive mucin overproduction and neutrophilic inflammation in vivo [147]. These derivative studies collectively indicate that SO_2_-formed acid species activate epithelial pro-inflammatory programs and compromise cell viability, although the field urgently needs more direct gaseous SO_2_ exposure models to confirm whether these derivative responses fully replicate the chemistry and biology of inhaled SO_2_.

#### 4.4.2. Animal Experiments

Animal studies characterizing the toxicological effects of SO_2_ and NO_2_ in asthmatic models are compiled in Table 4. In Kunming mice, whole-body inhalation of SO_2_ (7.84 or 28 mg/m^3^, 6 h/day, 7 days) produced oxidative damage in lungs and hearts that varied by concentration and sex, characterized by lipid peroxidation and altered antioxidant enzyme function [148]. In OVA-sensitized BALB/c mice, SO_2_ derivatives aggravated ovalbumin-induced asthma by targeting TRPV1 channels and disrupting tight junction proteins (occludin, claudin-1, and ZO-1), leading to increased vascular permeability, inflammatory cell infiltration, and AHR [149]. In OVA-sensitized Wistar rats, inhalation of SO_2_ (28 mg/m^3^, 1 h/day, 7 days) upregulated MUC5AC and intercellular adhesion molecule-1 (ICAM-1) expression in airway tissue, promoted inflammatory cell infiltration, and enhanced mucus secretion [150]. In another Wistar rat asthma model, SO_2_ exposure (5.6 mg/m^3^, 1 h/day, 7 days) activated NF-κB signaling and elevated TNF-α, IL-6, IL-4, and IgE levels, while promoting eosinophil and neutrophil recruitment, indicating dysregulated inflammatory and immune responses [151]. In OVA-sensitized C57BL/6 mice, SO_2_ inhalation (10 mg/m^3^, 30 min/day, 7 days) markedly amplified pulmonary pathological injury and increased eosinophil-rich leukocyte counts compared with OVA alone. Concurrently, MUC5AC, TNF-α, Th2 cytokines (IL-4, IL-5, and IL-13), and STAT6 expression were further upregulated, while SO_2_ exposure alone elevated STAT6 mRNA and hydrogen peroxide content in the lung [25].

The shared activation of Th2 cytokines and STAT6 by SO_2_ aligns closely with the effects of PM_2.5_ and NO_2_ described in previous sections, reinforcing the idea that diverse pollutants converge on common immune nodes. In clinical asthma, Th2 polarization and STAT6-driven IgE synthesis are hallmark features of allergic inflammation, and the experimental demonstration that SO_2_ derivatives target TRPV1 and disrupt tight junction proteins bridges animal toxicology to human airway pathology, because TRPV1 upregulation and epithelial barrier compromise are both documented in severe asthmatic airways [27].

### 4.5. MPs

#### 4.5.1. Cellular Experiments

In human lung epithelial A549 cells, polystyrene nanoplastics (PS-NPs; 60 nm, 10–500 μg/mL) were rapidly internalized within 3 h, accumulated in the cytoplasm, and induced dose-dependent cytotoxicity accompanied by ROS generation and mitochondrial membrane potential collapse [152]. Similarly, A549 cells exposed to nanoplastics of different sizes (50, 100, and 200 nm) and surface charges (neutral, positive, and negative) showed that smaller particles and positively charged particles exerted the strongest toxic effects, promoting epithelial-to-mesenchymal transition (EMT), increasing intracellular ROS, and disrupting mitochondrial function [153]. The responsiveness of airway epithelium to MPs appears compromised in asthma. In primary human nasal epithelial cells isolated from patients with asthma and COPD, exposure to polystyrene MPs (1–10 μm, 100 μg/mL) elicited a markedly blunted release of IL-6 and IL-8 compared with cells from healthy donors, indicating that pre-existing airway disease impairs the epithelial inflammatory response to microplastic stimulation [154].

#### 4.5.2. Animal Experiments

Animal experiments confirm that MPs induce pulmonary inflammation, oxidative stress, and aggravated airway dysfunction, particularly in asthmatic contexts. In Sprague–Dawley rats, intratracheal instillation of polypropylene nanoplastics (PP-NPs; 25 nm, 2 mg/kg) activated the p38 MAPK-mediated NF-κB pathway, caused mitochondrial damage, and increased TNF-α and IL-6 levels in lung tissue [96]. In C57BL/6 mice, intratracheal instillation of polystyrene MPs (PS-MPs; 10 μm, 0.5–50 μg per mouse, every two days for two weeks) impaired pulmonary physiology in normal mice and further aggravated AHR and inflammatory cell infiltration in OVA-sensitized asthmatic mice [97]. Transcriptomic studies further reveal the molecular breadth of MP-induced lung injury. In Sprague–Dawley rats, intratracheal instillation of PS-MPs (100 nm, 0.5–2 mg/200 μL, every two days for two weeks) caused alveolar destruction, bronchial epithelial disarray, and upregulated IL-6 and IL-1β [98]. In a whole-body inhalation model, Sprague–Dawley rats exposed to polystyrene micro(nano)plastics for 14 days showed concentration-dependent increases in lung tissue transforming growth factor-beta (TGF-β) and TNF-α expression, indicating inflammatory protein accumulation even at the organismal level [155].

The MP-activated nodes in the ROS and NF-κB signaling pathways directly overlap with those affected by conventional air pollutants, which helps explain why the health risks associated with concurrent exposure to both air particulate matter and plastic pollutants may be more severe than those resulting from exposure to either pollutant alone.

These experimental findings should be interpreted cautiously. The toxicological literature on MPs has relied predominantly on standard polystyrene microspheres whose physicochemical properties differ markedly from the heterogeneous mixtures encountered environmentally. Moreover, commonly employed non-inhalation exposure routes, such as tracheal instillation, poorly simulate the chronic low-dose inhalation patterns typical of ambient human exposure. These overlapping signaling nodes, ROS generation and NF-κB activation, are central to human asthmatic inflammation, suggesting that the pulmonary injury induced by MPs in animal models may aggravate existing airway disease in humans if similar exposure levels and particle characteristics occur [156].

### 4.6. PFAS

#### 4.6.1. Cellular Experiments

In vitro evidence demonstrates that PFAS directly injures respiratory epithelial cells through oxidative stress, apoptosis, and inflammatory signaling. In human lung adenocarcinoma A549 cells, perfluorooctane sulfonate (PFOS; 50–400 μmol/L, 24 h) induced apoptosis in a dose-dependent manner, characterized by ROS accumulation, mitochondrial membrane potential collapse, and caspase-9/caspase-3 activation, indicating a ROS-mediated mitochondrion-dependent apoptotic pathway [157]. Concurrently, exposure of A549 cells to a mixture of perfluoroalkyl acids (PFOA, PFOS, perfluorononanoic acid (PFNA) and PFDA) triggered protective mitochondrial and endoplasmic reticulum autophagy, p62 degradation, and enlarged autophagosomes, suggesting that lung cells mount an adaptive autophagic response to counteract PFAS-induced organelle damage [158]. In human bronchial epithelial BEAS-2B cells, a PFAS mixture containing PFOA, PFOS, and perfluorobutanoic acid (PFBA) increased caspase-1 secretion and upregulated steady-state mRNA levels of IL-6, IL-5, and IL-8, indicating activation of inflammatory and pyroptotic pathways [26].

Beyond lung-specific models, short-chain PFAS including hexafluoropropylene oxide dimer acid (HFPO-DA), PFBA, perfluorobutanesulfonic acid (PFBS), and perfluorohexanoic acid (PFHxA) altered oxidative stress biomarkers in human liver, kidney, muscle, and microglia cell lines, indicating that PFAS broadly weaken cellular antioxidant defenses across multiple tissue types [159].

#### 4.6.2. Animal Experiments

In C57BL/6 mice, inhalation of perfluorooctanoic acid (PFOA; 0.1%, 6 h/day, 14 days) or dietary exposure (0.02%, 10 weeks) increased AHR to methacholine and elevated the number of pulmonary macrophages, indicating that chronic PFOA exposure impairs airway physiology even without allergic sensitization [160]. In male ICR mice, 21 days of PFOA exposure via drinking water (0–250 ppm) altered T lymphocyte subsets in the spleen and thymus. Notably, all doses decreased CD8+ lymphocytes in the spleen, while 50 and 250 ppm increased CD4+ lymphocytes, skewing the CD4/CD8 ratio. Concurrently, PFOA elevated splenic expression of pro-inflammatory cytokines TNF-α, IL-1β, and IL-6, demonstrating systemic immunomodulatory effects [161]. In adult C57BL/6 mice, sub-chronic PFOS exposure via gavage (0–10 mg/kg/day, 30 days) disrupted the balance of type 1 and type 2 cytokines. Specifically, PFOS dose-dependently decreased IFN-γ (a Th1 cytokine) while increasing IL-4 (a Th2 cytokine), shifting immune responses toward Th2 polarization [162].

Notably, the ROS, NF-κB, and inflammasome signals activated by PFAS are precisely the same pathways amplified by PM_2.5_, O_3_, and NO_2_. When these pollutants coexist, the lung faces simultaneous hits on shared molecular targets, which is why real-world mixed exposures carry greater asthma risk than laboratory single-agent studies suggest.

**Figure 6:**
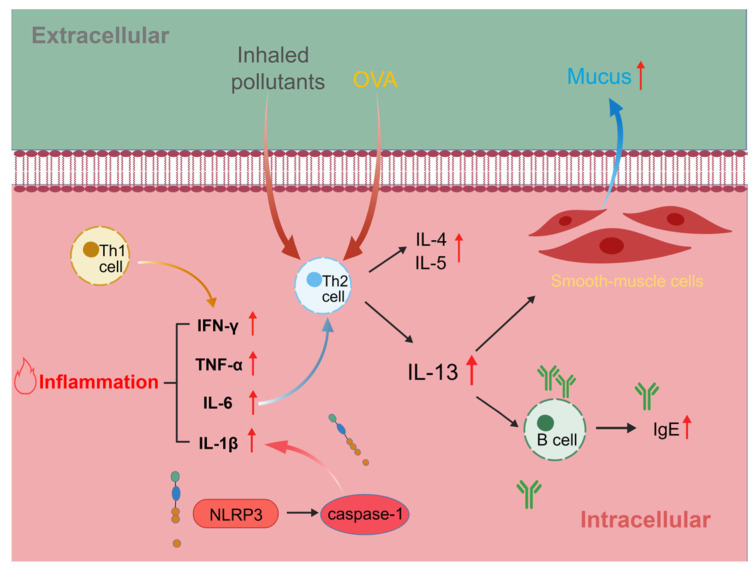
Schematic diagram illustrating the immunological mechanisms by which inhaled air pollutants drive airway inflammation in asthma. Inhaled pollutants and OVA promote Th2 cell polarization, leading to increased IL-4, IL-5, and IL-13 secretion. IL-13 drives B-cell IgE production and smooth muscle mucus hypersecretion, while Th1-derived IFN-γ is suppressed, shifting the Th1/Th2 balance toward Th2 dominance. Concurrently, NLRP3 inflammasome activation triggers caspase-1-mediated IL-1β maturation, accompanied by elevated TNF-α and IL-6, collectively amplifying airway inflammation. (T helper type 2 cell (T2); Interleukin-4 (IL-4); Interleukin-5 (IL-5); Interleukin-13 (IL-13); Interleukin-6 (IL-6); Immunoglobulin E (IgE); T helper type 1 cell (Th1); Interferon-gamma (IFN-γ); NOD-like receptor family, pyrin domain containing 3 (NLRP3); Cysteine-aspartic acid protease-1 (Caspase-1); Interleukin-1 beta (IL-1β); Tumor necrosis factor-alpha (TNF-α); Ovalbumin (OVA)). The red upward arrow indicates that the indicator rises.

Several experimental constraints limit the translational confidence of these PFAS data. Research has focused primarily on a few long-chain compounds, notably PFOA and PFOS, while toxicity data for the growing number of short-chain and novel alternatives remain limited. Furthermore, humans are simultaneously exposed to multiple PFAS, and how these mixtures interact to affect respiratory health requires further investigation. The convergence of PFAS-activated pathways on ROS, NF-κB, and inflammasome signaling aligns with established human asthmatic endotypes characterized by oxidative and neutrophilic inflammation, lending mechanistic plausibility to the epidemiological associations between perfluoroalkyl exposure and asthma risk reported in the earlier discussion [163]. The convergent immunological mechanisms by which inhaled air pollutants drive Th2 polarization, NLRP3 inflammasome activation, and downstream airway inflammation are schematically illustrated in Figure 6.

## 5. Research on Combined Effects (Air Pollutants and Temperature) on Asthma

### 5.1. Extreme Temperatures

The impact of thermal conditions and ambient factors on asthma pathogenesis has become a focal point in contemporary respiratory research. Across the globe, the frequency and intensity of extreme weather events are increasing, revealing marked regional heterogeneity. This variation in temperature and environmental conditions can significantly affect asthma.

In environmental epidemiology, extreme heat is commonly defined as periods when daily mean temperature exceeds the 95th percentile of the local historical distribution for at least two consecutive days, with extreme cold falling below the 5th percentile [164]. A health-based definition using the Universal Thermal Climate Index indicates that three consecutive days with night-time minimum UTCI ≈ 15 °C and daytime maximum UTCI ≈ 34.5 °C (both at the 95th percentile) are the best predictors of excess mortality attributable to heat [165].

### 5.2. Effects of Extreme Temperature on Asthma

#### 5.2.1. Epidemiological Studies on Extreme Temperatures

A 2022 systematic review and meta-analysis registered with PROSPERO quantified the independent respiratory impacts of extreme weather events, pooling data from multiple continents. The analysis found that extreme heat and cold collectively increased the risk of asthma events by 1.18-fold (95% CI: 1.13–1.24), asthma hospital admissions by 1.10-fold (95% CI: 1.04–1.17), and asthma mortality by 2.10-fold (95% CI: 1.35–3.27), with children and females identified as disproportionately vulnerable subgroups [166]. This meta-analytic evidence provides the population-level foundation for asserting that thermal extremes are independent risk modifiers of asthma morbidity.

A time-series analysis in Hong Kong demonstrated that warm-season temperature rises were associated with surges in asthma-related hospitalizations, particularly when combined with high humidity [167]. Conversely, longitudinal data from eighteen Chinese cities revealed that cold temperatures (3 °C and −7 °C) increased the risk of acute asthma exacerbation by 68% and 73%, respectively, within a 0–2 day lag window [168]. In Atlanta, warm-season maximum temperatures showed a statistically significant correlation with emergency department visits for asthma [169]. These findings confirm that thermal extremes are not merely passive background conditions but active stressors that perturb airway homeostasis. Selected epidemiological investigations quantifying the temperature–asthma association across diverse climatic zones are summarized in Table 5.

#### 5.2.2. Toxicological Studies on Extreme Temperatures

At the toxicological level, extreme temperatures exert divergent but complementary effects on airway inflammation. In BALB/c mouse models, high temperatures (38–40 °C) aggravated allergic airway inflammation through upregulation of TRPV1 and amplification of Th2 cytokine responses [36]. Recent evidence further indicates that TRPV1-expressing nociceptor neurons drive airway neurogenic inflammation via substance P-dependent macrophage M1 polarization, establishing a neuroimmune axis for heat-induced inflammatory amplification [170]. In contrast, repeated exposure to temperature variation (simulating unstable weather) exacerbated airway inflammation via TRPA1 activation, a key mediator of neurogenic inflammation that can be attenuated by TRPA1 antagonists [37]. Cold air (4 °C) activates epithelial TRPA1 to induce thymic stromal lymphopoietin (TSLP) release and type 2 innate lymphoid cell expansion, revealing an epithelial–innate immune axis for cold-triggered exacerbation independent of neuronal signaling [171].

Collectively, these studies establish TRPV1 and TRPA1 as molecular sensors through which thermal stress independently initiates inflammatory cascades via neuronal and epithelial routes, providing the mechanistic entry point for pollutant–temperature interactions.

#### 5.2.3. Allergen-Mediated Modulation of Temperature Effects

Temperature extremes also modulate asthma risk indirectly by altering the abundance, seasonality, and allergenicity of biogenic aeroallergens. House dust mites (*Dermatophagoides farinae* and *D. pteronyssinus*), a major indoor trigger for perennial allergic asthma, exhibit temperature-dependent population dynamics with optimal growth at approximately 28 °C under controlled laboratory conditions [172]. Field studies confirm that indoor thermal conditions within 18–25 °C create favorable environments for dust mite proliferation and allergen accumulation [173].

Outdoor aeroallergens are subject to similar climate-driven shifts. A continent-wide analysis of 60 North American pollen-monitoring stations spanning 821 site-years between 1990 and 2018, integrated with Earth system model simulations, found that pollen seasons have lengthened by approximately 20 days and concentrations have increased by 21% over the study period; formal detection-and-attribution analysis indicated that anthropogenic climate forcing contributed roughly 50% (interquartile range: 19–84%) of the observed season lengthening and about 8% (interquartile range: 4–14%) of the concentration increase [174]. Ragweed (*Ambrosia artemisiifolia*), a principal autumn asthma trigger, shows particularly strong responses to elevated temperature and atmospheric CO_2_. In controlled-environment greenhouse experiments, doubling atmospheric CO_2_ concentration increased ragweed pollen production by 61% (*p* = 0.005) [175]. Elevated temperature amplifies this effect: at 26–30 °C, ragweed growth rate, male flower proportion, and pollen production are significantly higher than at lower temperatures, with concurrent upregulation of allergenic protein synthesis [176]. Long-term ecological monitoring confirms that warming has lengthened the ragweed pollen season across central North America in proportion to latitude-dependent temperature increases [177], while European records show that higher summer and autumn temperatures directly prolong ragweed flowering and delay pollen season termination [178].

Temperature extremes activate an allergen-mediated exacerbation pathway through the proliferation of indoor dust mite populations and the shift of outdoor pollen seasons toward earlier onset, longer duration, and greater intensity, with ragweed representing the most prominent example. This pathway operates concurrently with pollutant-driven oxidative and inflammatory toxicity. An integrated assessment of asthma risk under climate change must therefore account for this dual mechanism: direct thermo-chemical airway injury plus indirect allergen amplification.

**Table 5:** Epidemiological study about relationship between temperature and asthma.

Temperature	Location	Result	Reference
Heat	Atlanta	Emergency department visits for asthma showed statistically significant correlation with elevated warm-season maximum temperatures.	[169]
Heat	Hong Kong	Asthma-related hospitalizations surged in response to combined heat and humidity exposure during warm seasons.	[167]
Heat	Brazilian cities	The heat–asthma connection exhibited greater effect magnitudes in disadvantaged urban areas.	[179]
Cold	Korea cities	Exposure to 2 °C ambient temperature demonstrated a significant positive association with asthma hospitalization risk.	[180]
Cold	London	Subzero exposures (≤0.0 °C) demonstrated significant positive associations with asthma-related emergency service utilization.	[181]
Cold	Chinese cities	People exposed to low temperatures (3 °C and −7 °C) had a 68% and 73% increased risk of acute exacerbation of asthma within 0–2 days of lag.	[168]

Note: Extreme heat and cold are defined according to the 95th and 5th percentiles of local historical temperature distributions, respectively [164].

### 5.3. Synergistic Effects of Temperature and Air Pollutants

Population-level surveys have reported that the respiratory toxicity of air pollutants may be amplified under concurrent thermal stress, yet the cellular and systemic substrates for this amplification remain only partially clarified. The following experimental evidence provides a preliminary mechanistic counterpart.

In OVA-sensitized BALB/c mice, co-exposure to high temperature and NO_2_ aggravated AHR, inflammatory infiltration, and oxidative stress beyond the levels observed with either stressor alone, a pattern consistent with additive or synergistic interaction. Serum total IgE, IL-4, IL-6, TNF-α, ROS, and MDA increased, while IFN-γ and GSH decreased; notably, the TRPV1 antagonist capsazepine significantly attenuated these co-exposure effects, suggesting TRPV1-mediated neurosensory activation as a plausible convergence point for heat and NO_2_ toxicity, though causality in humans remains to be established [182]. Complementing this, co-exposure to high humidity and NO_2_ further upregulated TRPV1, TRPA1, and TRPV4 protein expression in lung tissue, amplified oxidative stress, and intensified Th2-polarized inflammation compared with NO_2_ alone, indicating that thermal stress may prime TRP channels for exaggerated responses to oxidant gases [183]. In allergic airway models, concurrent O_3_ and high temperature disrupted nasal epithelial tight junctions and induced nasal microbiota dysbiosis more severely than either exposure alone; although this study employed allergic rhinitis rather than asthma, the shared epithelial barrier pathology supports the generalizability of the heat–O_3_ barrier disruption mechanism to lower airways, pending direct asthma-specific confirmation [184]. Cold stress creates a distinct but complementary susceptibility substrate through persistent immune and epigenetic reprogramming. In C57BL/6J mice, childhood co-exposure to cold stress and PM_2.5_ led to persistent alveolar wall thickening, Th2/Th1 imbalance, impaired Th17/regulatory T cell (Treg) differentiation, and elevated OVA-specific IgE in adulthood, indicating that early-life thermal–pollutant interactions may imprint long-lasting asthma susceptibility [185]. Mechanistically, combined cold and PM_2.5_ exposure increased histone H3K9 and H3K14 acetylation at the IL-4 gene promoter in CD4^+^T cells, suggesting that cold stress and particulate matter converge on epigenetic machinery to skew immune responses toward Th2 polarization [186]. Beyond these established pollutant–temperature pairs, our recent work investigated the combined effect of high temperature (40 °C) and traffic-source PM_2.5_ on allergic airway inflammation in BALB/c mice. Both stressors individually aggravated AHR, and worsened airway histopathology through oxidative stress and TRPV1 activation. However, no synergistic interaction was observed between 40 °C and 100 μg/m^3^ traffic–PM_2.5_ under the tested conditions. Pharmacological blockade with the TRPV1 antagonist capsazepine or vitamin E effectively attenuated airway inflammation, suggesting tractable therapeutic targets [187].

Collectively, these converging lines of evidence suggest that pollutant–temperature interactions may reflect biologically plausible phenomena with identifiable molecular entry points, although the statistical robustness of synergy versus additivity remains to be fully established across environmentally realistic exposure regimes. Epidemiological patterns of amplified asthma risk under thermal extremes, as outlined in these studies, are paralleled by animal models showing TRPV1-dependent neuroimmune amplification, epigenetic Th2 skewing and epithelial barrier failure, while cellular studies trace these effects to ROS generation, TRP channel gating and histone acetylation. An integrated framework that links population surveillance to pathway-level toxicology represents a promising next step toward translating these findings into real-world asthma care, yet it should be regarded as an emerging model rather than a confirmed paradigm.

Increased temperatures appear to mediate rapid-onset inflammation via TRPV1 neurosensory activation and acute oxidative injury, whereas cold exposure may promote airway remodeling through persistent immune imbalance and sustained oxidative stress. These findings indicate that extreme temperatures could jointly exacerbate asthma through multiple pathways, though the relative contribution of each pathway in human populations requires further delineation. Therefore, it is crucial to develop targeted interventions based on the hypothesized interaction mechanisms between temperature and pollutants to control asthma, while acknowledging that current evidence derives primarily from controlled laboratory settings.

**Figure 7:**
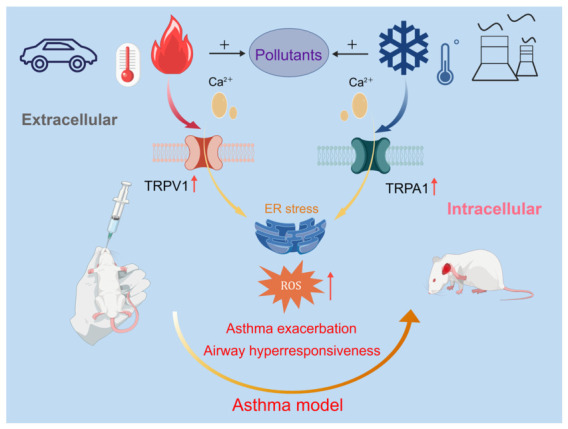
Schematic diagram illustrating the synergistic mechanisms of pollutant–temperature co-exposure in aggravating asthma. Extreme heat and cold temperatures, in combination with airborne pollutants, respectively activate TRPV1 and TRPA1 ion channels, provoking intracellular calcium (Ca^2+^) overload. The subsequent ER stress and ROS accumulation exacerbate AHR and asthma deterioration in murine models. (Transient receptor potential vanilloid 1 (TRPV1); Transient receptor potential ankyrin 1 (TRPA1); Endoplasmic reticulum (ER); Reactive oxygen species (ROS)). The red upward arrow indicates that the indicator rises.

Despite increasing attention to synergistic effects, the current evidence remains methodologically limited. Most co-exposure studies have employed two-factor designs that do not reflect the multi-pollutant complexity of real-world environments. In addition, animal protocols have typically used acute, high-dose regimens that poorly simulate the chronic, low-dose exposures characteristic of human populations. Consequently, the synergistic framework proposed here, while biologically plausible, should be regarded as an emerging model awaiting confirmation from long-term, environmentally realistic studies. The integrated molecular framework through which extreme temperatures and air pollutants synergistically aggravate asthma via TRPV1/TRPA1 activation, intracellular calcium overload, and ER stress is depicted in Figure 7.

## 6. Conclusions

### 6.1. Future Perspectives

This review assembles evidence suggesting that interactions between extreme temperatures and air pollutants may exacerbate asthma pathogenesis more severely than either factor alone, thereby offering a perspective that complements traditional single-factor frameworks without supplanting them.

Climate-sensitive respiratory risks emerged from thermo-chemical synergies between thermal extremes and airborne pollutants. Both high-temperature (e.g., 40 °C) and low-temperature conditions potentiated asthmatic pathogenesis via TRPV1-dependent airway hyperreactivity, redox imbalance, and cytokine cascades, whereas PM_2.5_, O_3_, SO_2_, NO_2_, MPs and PFAS aggravated neutrophilic inflammation and pulmonary mechanical dysfunction. These environmental factors may act synergistically under combined exposure scenarios, potentially exacerbating oxidative damage, although the extent of synergy in real-world human populations remains to be quantified. Effective mitigation therefore requires multifaceted intervention strategies: advanced environmental surveillance systems, low-emission urban design (green infrastructure deployment, clean transportation networks), precision pollution mitigation protocols (vehicle access regulation, renewable energy integration), and geographically adaptive interventions (community respiratory protection programs, clean heating solutions in northern China, ozone precursor management in southern regions). Concurrent deployment of climate-resilient policies and precision medical interventions may attenuate climate-sensitive respiratory disease burden, pending further validation of interaction thresholds in diverse populations.

Future research should focus on elucidating multiscale mechanistic interactions between air pollutants and temperature to uncover their interactive toxicity pathways under climate change.

Population heterogeneity requires mechanistic dissection of exposure–response dynamics in vulnerable subgroups: children (alveolar developmental windows), the elderly (immunosenescence), and pregnant women (placental barrier permeability). These findings would guide the design of advanced respiratory protection technologies (e.g., pediatric 3D-printed, anatomically fitted respirators) and targeted behavioral protocols (e.g., gestation-specific outdoor activity optimization).

Technological innovation demands integration of advanced in vitro models—particularly dynamic air–liquid interface exposure systems coupled with pulmonary organoids to model human-relevant toxicity pathways. Single-cell multiomics integration will delineate pollutant-induced transcriptomic/epigenetic perturbations, while patient-derived organoid platforms could facilitate personalized toxicity mapping, thereby guiding region-specific pollution remediation and precision prophylactic development. Translating animal-derived insights into human organoid models will accelerate decoding of human pathophysiology.

Establishing a unified “environmental surveillance–health early warning–policy intervention” framework becomes essential, integrating real-time monitoring with responsive governance mechanisms. Internet of Things (IoT)-enabled asthma risk prediction systems, artificial intelligence (AI)-optimized urban ventilation corridors, and geographically tailored governance measures (e.g., northern China’s clean heating initiatives versus southern ozone precursor co-reduction strategies) should be prioritized for deployment. Global harmonization of extreme climate-pollutant exposure metrics via unified data platforms empowers transnational asthma prevention efforts, advancing understanding of mechanisms, individualized protection, and planetary-scale environmental health governance.

### 6.2. Limitations

Additional methodological and evidentiary limitations have been discussed in the relevant sections above. In summary, this narrative synthesis did not undertake systematic quantitative evaluation through meta-analysis, nor did it fully delineate the mechanistic underpinnings of population-specific susceptibility disparities. Stronger claims regarding combined exposures should be interpreted accordingly.

## Figures and Tables

**Table 1:** Selected epidemiological studies on the relationship between air pollution and asthma.

Pollutants	Country/Region	Study Design	Number	Ages	Year	Main Finding	Reference
PM_2.5_, O_3_, SO_2_, NO_2_	China	Case-crossover	2507	0–18	2017	NO_2_ played an important role, but O_3_ had no significant effect.	[113]
PM_2.5_, O_3_	USA	Case-crossover	21854	3–17	2014	Short-term O_3_ was associated with an increased asthma risk in children.	[114]
NO_2_	Japan	Time-series	3427	0–12	2010	Higher NO_2_ levels increased asthma hospitalizations in children (<12).	[115]
PM_2.5_, NO_2_	USA	Time-series	3503	1–65	2011	Dust events increased all-age asthma hospitalizations.	[116]
SO_2_	Canada	Cohort	1842	2–4	2009	Short-term SO_2_ increased asthma frequency in nearby children.	[117]
O_3_	USA	Time-series	121621	0-≥65	2014	Short-term O_3_ exposure and asthma visits varied by level of urbanization.	[56]
SO_2_, PM_2.5_	Canada	Case–control	429	0–4	2013	Longer residence near aluminum smelters raised asthma hospitalization risk.	[118]
PM_2.5_, O_3_, NO_2_	Japan	Case-crossover	1447	0–14	2015	O_3_ was associated with asthma attacks requiring visits to primary care facilities.	[119]
PFAS	Taiwan (Region)	Case–control	456	10–15	2016	Association between serum PFAS and asthma.	[102]
PFAS	Norway	Cohort	675	16–18	2019	Exposure to PFAS in adolescents was positively associated with asthma risk.	[15]
PFAS	USA	Case-sectional	456	12–19	2014	An association between PFAS exposure and asthma in American children.	[103]

**Table 2:** Research of animal experiments about the effect of PM_2.5_ on asthma.

Animal Model	Results	Method	Exposure	Reference
BALB/c mice	Activates TRPA1/TRPV1, inducing NGF/SP release and neurogenic inflammation.	Intratracheal instillation	1.6 or 8.0 mg/kg; 28 or 42 days	[125]
BALB/c mice	Activates TLR2/TLR4/My88D, driving allergic lung inflammation.	Intratracheal instillation	0.1 mg/mouse; 4 times at two-week intervals	[126]
C57BL/6 mice	Downregulates Kdm6a, increasing H3K4/H3K9 methylation at IL-6/IFN-β promoters, altering immune response.	Nasal inhalation	10 or 100 mg/kg; once daily for 30 days	[124]
Swiss mice	Increases inflammatory cytokines, eosinophils, and mucin 5AC (MUC5AC), decreases IFN-γ.	Intratracheal instillation	1.58 or 4.98 mg/kg; 7 times at 1-day intervals	[127]
Asthmatic mice	Activates Notch, worsening Th1/Th2 imbalance and multi-pathway immune disruption.	Inhalation method	510 μg/m^3^; 4 times daily (15 min/session, 0.5 h intervals) for 30 days	[128]

Note: transient receptor potential ankyrin 1 (TRPA1); transient receptor potential vanilloid 1 (TRPV1); nerve growth factor (NGF); substance P (SP); Toll-like receptor 2/4 (TLR2/4); myeloid differentiation primary response 88 (MyD88); lysine demethylase 6a (Kdm6a); histone H3 lysine 4/9 (H3K4/H3K9); interleukin (IL); interferon (IFN); mucin 5AC (MUC5AC); T helper type 1/2 cell (Th1/2).

**Table 4:** Research of animal experiments about the relationship between SO_2_/NO_2_ and asthma.

Animal Model	Results	Method	Exposure	Reference
Wistar rats	Activates NF-κB, promoting TNF-α/IL-6 and elevating IL-4/IgE, exacerbating OVA-induced pulmonary inflammation and asthma.	Inhalation method	5.6 mg/m^3^; 1 h/day for 7 days	[151]
C57BL/6 mice	Causes modest airway injury and exacerbates OVA-triggered Th2 lung inflammation via STAT6.	Inhalation method	10 mg/m^3^; 0.5 h/day (30 min/day) for 7 days	[25]
Wistar rats	Induces inflammatory cell infiltration, mucus hypersecretion, and collagen deposition in OVA-sensitized rat lungs, exacerbating allergic airway inflammation.	Inhalation method	2 or 5 mg/m^3^; 5 h/day for 28 or 42 days	[141]
C57BL/6 mice	Potentiates allergic airway inflammation with elevated ROS/MDA and depleted GSH.	Inhalation method	25 ppm; 6 h/day for 3 or 5 days	[143]

Note: nuclear factor kappa-B (NF-κB); tumor necrosis factor-alpha (TNF-α); interleukin (IL); immunoglobulin E (IgE); signal transducer and activator of transcription 6 (STAT6); ovalbumin (OVA); reactive oxygen species (ROS); malondialdehyde (MDA); glutathione (GSH).

## Data Availability

No new data were created or analyzed in this study. Data sharing is not applicable to this article.
